# On the limitations of closed-loop geothermal systems for electricity generation outside high-geothermal gradient fields

**DOI:** 10.1038/s44172-025-00458-7

**Published:** 2025-07-01

**Authors:** Sri Kalyan Tangirala, Víctor Vilarrasa

**Affiliations:** 1 Global Change Research Group (GCRG), IMEDEA-CSIC-UIB, Esporles, Spain; 2 Department of Civil and Environmental Engineering (DECA), Universitat Politècnica de Catalunya·BarcelonaTech (UPC), Barcelona, Spain

**Keywords:** Hydrology, Energy supply and demand

## Abstract

Closed-Loop Geothermal Systems (CLGS) involve connecting the injection and production wells through several borehole-sized parallel laterals instead of circulating a working fluid through a fracture network. Companies have garnered millions of dollars in investments on the claim that CLGS is truly scalable for both heating and electricity generation purposes. We show that high flow rates in the laterals lead to a steep drop in production temperatures because of a rapid cooling of the rock matrix surrounding the wells. Overcoming this physical limitation of CLGS demands an expensive task of drilling several multilaterals to reduce the lateral flow rate. Yet, simulation results indicate that, for a reservoir temperature of 180 °C, the total revenue of these systems fail to recover the lifetime costs incurred, even with 30 multilaterals and a production rate of 75 kg/s, which clearly indicates that CLGS are not scalable for solely electricity generation.

## Abstract (web summary)

Sri Kalyan Tangirala and Víctor Vilarrasa report that limited conduction heat exchange and high drilling costs result in poor economic returns for electricity generation in closed-loop geothermal systems outside high geothermal gradient fields. This questions industry claims of scalability.

## Introduction

Geothermal energy is a renewable energy resource that is extremely abundant and non-intermittent in nature, which can sustainably cater both our heating and electricity needs^1–3^. Electricity generation from hydrothermal systems is the most mature of all geothermal technologies as it has been active for over a century now, but these systems are limited to tectonic plate boundaries and regions comprising geothermal hotspots^4^. Exploiting the fact that we can harness geothermal energy from anywhere on the Earth if we reach depths with temperatures above 150 °C, the concept of Enhanced Geothermal Systems (EGS) was introduced, where we target deep crystalline rock (3 to 6 km depth)^5^. At these depths, rock permeability is usually insufficient to establish good connectivity between the injection and production wells, which requires hydraulic stimulation of pre-existing fractures or creation of new ones to enhance permeability. These processes induce swarms of microseismicity in the target reservoir, where the magnitude is usually limited to *M*_w_ < 2 (permissible limit as it is rarely felt on the surface), but a few events of higher magnitude (e.g., *M*_w_ 3.4 at Basel, Switzerland and *M*_w_ 5.5 in Pohang, Korea Republic) have caused damage and alarmed the public and investors^6,7^. Additionally, initial EGS projects also had a problem of inability to establish high flow rates, which resulted in poor economics of the projects^8^.

**Fig. 1:**
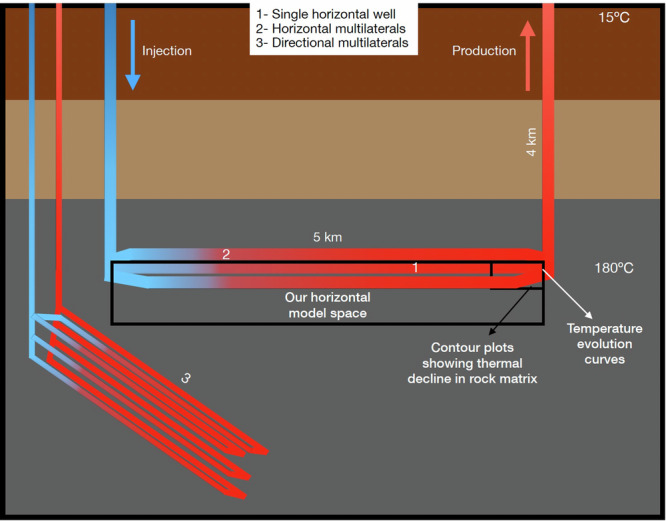
Sketch of different types of closed-loop geothermal systems. Injection and production wells can be connected by a (1) single horizontal well, (2) multiple horizontal wells or multilaterals, and (3) directional multilaterals as labeled in the figure. Basic parameters used in our study, like the depth and length of multilaterals and the surface and reservoir temperatures, are shown in the figure. Solid black lines mark our horizontal model space, and the small rectangular compartment near the end of the horizontal well is where we make the temperature contour plot for Fig. 2. Dimensions in the figure are not to scale.

Closed-Loop Geothermal Systems (CLGS) have been conceptualized in an attempt to find alternative ways of efficiently extracting scalable deep-geothermal energy that would not have the risk of inducing seismicity^9^. CLGS involve connecting the injection and production wells by drilling wellbore-sized horizontal or directional ‘laterals or loops’ (Fig. 1). This concept has been successfully tested through a pilot plant established in Alberta, Canada, by Eavor Technologies in 2019 that is currently producing some amount of hot water (not for electricity generation)^10^. There are industry claims of scalable electricity generation using CLGS, but there is no power plant that has proven this capability of CLGS to date. Most of the studies on CLGS that show the long-term (30–40 years) performance of these systems suggest that they are good for heat production, but not efficient for electricity generation, owing to thermal losses, but they do not elaborate the reasons for it, which is a key aspect of our work^9,11,12^.

Wellbore heat transfer during injection and production of fluids has been observed and documented since the 1960s^13,14^. On one hand, the heat exchange in the injection wellbore is strongly dependent on the injection temperature rather than the surrounding geothermal gradient when a cold fluid is injected^14,15^. On the other hand, in the production well, even though the temperature drop is lower than the geothermal gradient, it is significant and is inversely proportional to the fluid flow rate, because lower flow rates have more contact time with the surrounding rock and lead to higher dissipation of heat^14,16^. CLGS have very long open-hole or cased horizontal or directional sections, which act as the only fluid contact zones with the rock matrix for heat transfer, and the effective contact area with the rock matrix is less than that of fracture-based EGS (Fig. 1)^17^. The temperature of the produced fluid is a cumulative result of heat losses from the horizontal laterals and production well sections (10 s of °C) of the borehole and is dependent on factors such as reservoir temperature, flow rates, length and number of laterals, rock thermal conductivity, among others^9,11,12,18^.

Even in reservoirs with high temperatures (*T* > 200 °C), many long multilaterals would need to be drilled to compensate for the thermal losses and reach megawatt scale of electricity generation^11,19^. Millions of dollars in funding have been secured by companies that promise scalable baseload electricity from CLGS^20^. For example, Eavor 2.0, a concept proposed by Eavor Technologies, involves drilling around 82 km of open-hole directional multilaterals in very hot crystalline rock for electricity generation, something that has never been done before^19^. In this study, we focus on the electricity generation capability of CLGS in granites over a short term of one year for a reservoir temperature of 180 °C, while varying the flow rates and number of laterals (Type 2 in Fig. 1). We show the rapid thermal decline in the rock matrix surrounding the wells and conclude that the large temperature drops by the time the fluid reaches the end of the horizontal section make these systems inefficient for electricity generation in regions with average or even above average geothermal gradients, which seriously question the universal scalability of these systems. We combine our modeling results with cost-revenue analysis of CLGS and find that the return on investment (ROI) is negative for all our cases, implying that CLGS is not scalable for electricity generation, even with a significant advancement in drilling technologies that would drive the drilling costs down.

## Methodology

### Governing equations

Our fully-coupled Thermo-Hydraulic (TH) simulations are governed by solving the mass balance of water

1 $$\frac{\phi }{{K}_{f}}\frac{\partial p}{\partial t}+\nabla \cdot {{{\bf{q}}}}={f}_{w}$$

where *ϕ* [-] is porosity, *K*_*f*_ [ML^-1^ T^-2^] is water bulk modulus, *p* [ML^-1^T^-2^] is fluid pressure, *t* [T] is time, **q** [L^3^T^-1^] is the water flux, given by Darcy’s law, and *f*_*w*_ is the sink/source term. The energy balance equation is given as

2 $$\frac{\partial ({e}_{s}{\rho }_{s}\,(1\,-\phi )\,+\,{e}_{l}{\rho }_{l}\phi )}{\partial t}\,+\nabla \,\cdot \,\left({{{{\bf{i}}}}}_{c}\,+\,{{{{\bf{j}}}}}_{{es}}\,+\,{{{{\bf{j}}}}}_{{el}}\right)={f}^{Q}$$

where ${e}_{s}$ and ${e}_{l}$ are the internal energy of the solid and liquid, respectively, ${{{{\bf{i}}}}}_{c}$ is the energy flux due to conduction through the porous medium, ${{{{\bf{j}}}}}_{{es}},\,{{{{\bf{j}}}}}_{{el}}$ are advective fluxes of energy in the solid and liquid phases caused by mass motions, ${\rho }_{s}$, ${\rho }_{l}$ and are the solid and liquid densities, and ${f}^{Q}$ is an internal/external heat supply term.

The power output of CLGS is computed as

3 $$P=\dot{m}\cdot {C}_{p}\cdot \left(T-{T}_{o}\right) \cdot \eta$$

where *P* is the power output, *ṁ* is the mass flow rate of the fluid (in kg/s), *C*_*p*_ is the specific heat capacity of water at constant pressure (4200 J kg^-1^ °C^-1^), *T* is the production temperature, *T*_*o*_ is the base temperature of 100 °C below which electricity cannot be generated and *η* is the electricity generation efficiency. This efficiency is defined as the ratio of the net electric power that can be generated to the heat extracted from the reservoir (MW_e_/MW_th_), which ranges from 10-17%, with a world average of 12% based on the data from existing steam-based power plants as of 2014, while data from binary plants indicate efficiencies from 2% to 10%^21^. As steam cannot be produced from CLGS, we use an efficiency of 8% (depends on the inlet temperature of fluid into the generator), assuming a binary-power plant, as our production temperatures are less than 160 °C in most cases^21^.

The year-end revenue of the project is calculated using the Year-End Power (YEP) because the power output undergoes a steep decline from the maximum, and it can only consistently generate the YEP throughout the year. YEPs are calculated by using the Year-End Temperatures (YETs, that are the production temperatures at the end of the year) in Eq. 3. Considering various wholesale prices of electricity (6.4, 12.6, 16, 25, and 35 c/kWh), the revenue generated by the end of the year is computed by

4 $${Rev}={price} \cdot {YEP} \cdot 24 \cdot 365$$

where *Rev* is the yearly revenue. As the power output curves undergo a steady decline even beyond the first year of production (due to drop in production temperatures), YETs are calculated from the lines that fit the temperature evolution curves of horizontal models for each horizontal flow rate beyond the first year until the thirtieth (Supplementary Fig. 1 and Supplementary Table 1).

These subsequent YETs are used to calculate the YEPs (in kW_e_) of all our models from the second year until the thirtieth year, which would be used to calculate the yearly revenue. All of them are summed up to get the estimated total lifetime revenues, which are subtracted by the total lifetime costs to get the net profit or loss of all cases for various electricity prices and lateral drilling costs.

### Geometry and initial and boundary conditions

**Fig. 2:**
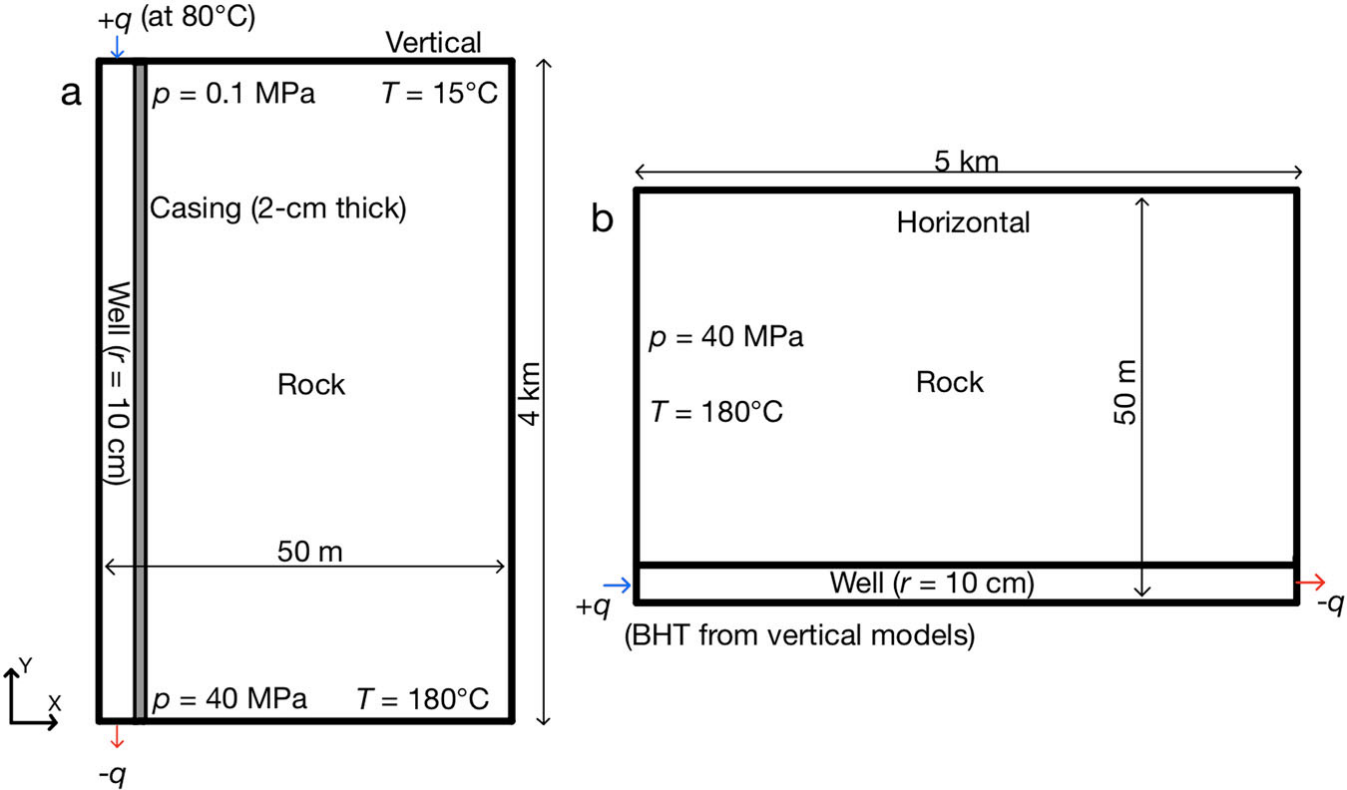
Geometry, initial and boundary conditions of our models. **a** Vertical models, and **b** horizontal models. Dimensions are not to scale.

We use two separate sets of 2-D axisymmetric geometries for our vertical (injection well) and horizontal lateral models. The vertical section comprises a wellbore of radius 10 cm, a 2-cm thick conductive casing followed by a 49.88-m thick rock matrix, all of which are 4-km in length. The horizontal lateral geometry has a wellbore of 10-cm radius that is directly in contact with the rock matrix of 49.9 m in thickness and has a length of 5 km (Fig. 2). All the components of our models, such as the wellbore, casing and rock matrix, are considered as porous media with their properties shown in Table 1. As water is always flowing in the wellbores of our models, we assign the properties of water to the material.

**Table 1:** Material properties of the well, casing and rock matrix

Material properties	Well	Casing	Rock matrix
Permeability (${m}^{2}$)	$1\cdot {10}^{-6}$	$1\cdot {10}^{-25}$	$1\cdot {10}^{-18}$
Porosity ($-$)	$0.99$	0.005	$0.01$
Thermal Conductivity (W/m/K)	$0.675$	43.75	$3$
Specific heat capacity (J/kg/K)	$4186$	400	$960$
Density (kg/m^3^)	$1$000	8060	$2700$

The initial and boundary conditions for the vertical models comprise hydrostatic pressure, which starts with atmospheric pressure of 0.1 MPa at the surface and reaches 40 MPa at a depth of 4 km as we consider acceleration due to gravity, with a surface temperature of 15 °C and a reservoir temperature of 180 °C at the bottom (geothermal gradient of 41.25 °C/km). Our horizontal models are at a constant depth of 4 km, with conditions of constant pressure and temperature equal to 40 MPa (gravity is not considered) and 180 °C, respectively, along the entire domain (Fig. 2). We run four vertical models with surface (total) flow rates of 12.5, 25, 50, and 75 kg/s with a water injection temperature of 80 °C. The vertical models yield as output the bottom-hole temperature (BHT) evolution in each case, and we consider the BHT after 17 days (BHT drops slowly for longer times) as the fluid input temperature in our horizontal models: 90 °C, 86.2 °C, 83.47 °C and 82.3 °C for 12.5 kg/s, 25 kg/s, 50 kg/s and 75 kg/s, respectively. We assume that the horizontal laterals are at the same depth and have enough separation to avoid thermal interference. Hence, we inject a proportionally lower flow rate to the number of laterals in the horizontal models at the corresponding BHT from the vertical models to account for multiple laterals. For example, the BHT at 17 days of a surface flow rate of 12.5 kg/s is used as the input temperature for a flow rate of 2.5 kg/s (12.5 kg/s-5 laterals) in our horizontal model to account for five laterals. We run the vertical models just to obtain the BHTs, so all of the results we present in this work are from the horizontal models. We run a total of 14 horizontal models by varying the number of laterals for the four surface flow rates—12.5 kg/s (with 1, 2 and 5 laterals), 25 kg/s (with 1, 2, 3 and 4 laterals), 50 kg/s (with 4, 8 and 12 laterals) and 75 kg/s (with 3, 12, 15 and 30 laterals) (see Table 2). The horizontal flow rates are obtained by simply dividing the surface flow rates by the number of laterals. The injection rates in all the horizontal models are incrementally increased from 0 kg/s to their respective rates by the end of the first day of injection, which are then constant for the rest of the year. All our models simulate one year of water circulation along the closed loop, while six of them (one for each of the horizontal flow rates) simulate the whole lifetime of thirty years to determine the temperature decline for revenue calculations (Supplementary Table 1).

**Table 2:** Summary of flow rates used in our horizontal models

Vertical flow rate (kg/s)	Number of laterals	Horizontal flow rate (kg/s)
12.5	1, 2, and 5	12.5, 6.25, and 2.5, respectively
25	1, 2, 3, and 4	25, 12.5, 8.33, and 6.25, respectively
50	4, 8, and 12	12.5, 6.25, and 4.167, respectively
75	3, 12, 15, and 30	25, 6.25, 5, and 2.5, respectively

### Numerical model setup

Our TH models are numerically simulated using the finite-element reservoir simulation software CODE_BRIGHT^22,23^. The mesh for our vertical and horizontal models has a total of 8200 and 5375 elements, respectively. The mesh is refined around the boreholes. The size of the element next to the rock matrix in the vertical models is as fine as 10^-5 ^m × 40 m and the farthest element is of dimensions 3.36 m × 40 m, whereas for the horizontal models, the finest element has dimensions of 4·10^-3 ^m × 40 m and the largest element has a size of 4.26 m × 40 m.

Mesh sensitivity analysis has been performed for our horizontal models and the current setup is optimal for fast calculation without compromising on the intricacies of the results. The vertical models have more number of elements. The software CODE_BRIGHT uses an adaptive time-step based on convergence of the Newton-Raphson iterations. CODE_BRIGHT is an established software and has been verified with other codes like TOUGH-UDEC^24^. It has also been validated with experiments and field studies and provides a very close representation of underlying physical processes in porous media^25–30^.

## Model limitations

In our model setup, we consider the wellbore as a porous medium with a very high permeability and porosity. This limits us from modeling turbulent flow in the wellbore, which is more prevalent in higher flow rates, because coupling a wellbore simulator with a Thermo-Hydro-Mechanical (THM) reservoir simulator is challenging and is not supported by the software we use. Since it is not possible to use a 2-D axisymmetric geometry to model all parts of the CLGS, i.e., injection well, horizontal multilaterals and production well, we separately model the vertical and the horizontal sections of the system. We use the surface flow rate in the vertical models (12.5, 25, 50, and 75 kg/s) to calculate the bottom-hole temperature (BHT) evolutions in each case. Then, we consider the BHT at 17 days as the input temperature for our horizontal models. We assume that the horizontal laterals are at the same depth and have enough separation to avoid thermal interference. Hence, we distributed the flow rate injected at the surface proportionally to the number of laterals and use the corresponding BHT from the vertical models into the horizontal ones, e.g., BHT of a surface flow rate of 75 kg/s and a horizontal flow rate of 2.5 kg/s (75 kg/s-30 laterals) to account for 30 laterals, and so on. We also consider the temperature at the end of the horizontal section to be the production temperature, while ignoring the temperature losses in the production borehole (we have computed it with a test vertical model to be a maximum of 10 °C for lower flow rates like 12.5 kg/s). The assumptions of a constant injection temperature for the horizontal model (when actually the BHT of the vertical well reduces with time) and the production temperature being the temperature at the end of the horizontal lateral make our results to be conservative, while the real values of temperatures and power outputs, and subsequent revenues, would be lower than what we show. We did not consider the effect of directional multilaterals on the steep decline in production temperatures, but studies have shown that there is an early steep drop in temperature for high-lateral flow rates in directional multilateral setups as well^19^. The occurrence of the steep temperature drop mainly depends on the lateral flow rate and not on its inclination or even the temperature of the reservoir (beyond 150 °C). Finally, we run all our models for a period of one year and six of them for the entire lifetime of 30 years (Supplementary Fig. 1), yet we show the main temperature and power curves just for one year because the focus of this study is to explain the reason behind this rapid thermal decline through analyzing what happens inside the rock matrix and back it up with a cost-revenue analysis (using 30-year results). Future works could include running long-term 3-D THM simulations of deep CLGS in high-temperature reservoirs to quantify the magnitude of potential cooling-induced seismic events.

## Results

**Fig. 3:**
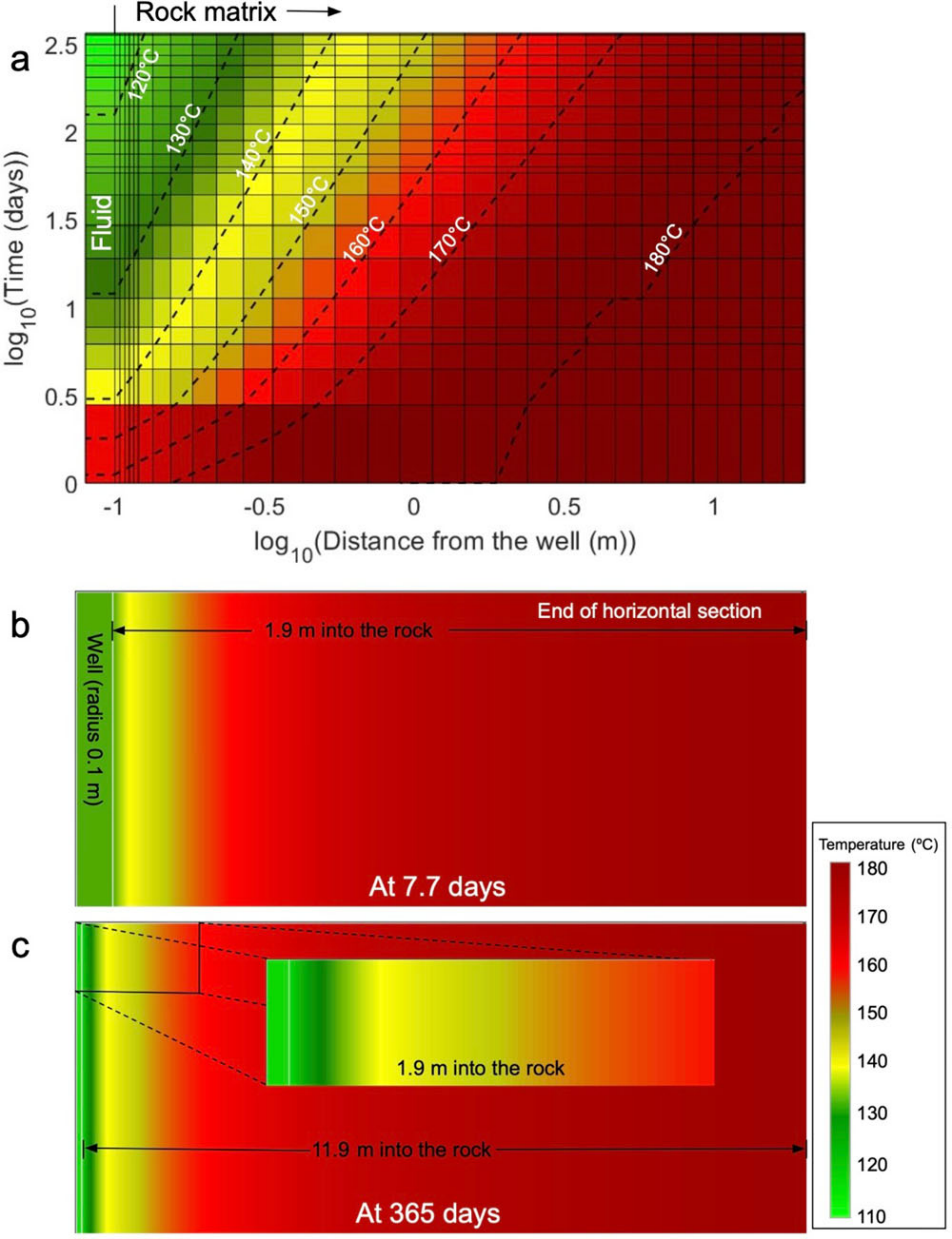
Contour plots showing the temperature distribution at the end of the horizontal lateral and rock matrix for the case of 25 kg/s-2 laterals. **a** The evolution of temperature for a 20-m section at the end of the horizontal lateral beginning from 0.08 m in the wellbore and extending into the rock matrix (19.9 m in (**a**), 1.9 m in (**b**), and 11.9 m in (**c**)). The small black vertical line on the top delineates the wellbore and the rock matrix sections in (**a**). The temperature decline in the rock matrix can be observed by the dotted temperature contour lines (isotherms). **b** Temperature distribution over the last section of the horizontal lateral at 7.7 days after the start of injection. **c** Temperature distribution over the last section of the horizontal lateral at the end of one year of injection.

Fluid injected into the closed-loop system (water in our case) gains heat as it goes down the vertical borehole. The attained temperature at the bottomhole is much lower than the bottom-hole temperature of the reservoir (180 °C in our case) because the circulating fluid rapidly cools down the rock surrounding the borehole, which prevents an effective heat flow for heating up the fluid. Subsequently, as the fluid initially passes through the horizontal loop, it absorbs heat from the interface with the surrounding reservoir rock and, at early times, reaches the ambient temperature of the rock before reaching the production well. However, continuous fluid flow through the horizontal loop depletes the heat from the rock interface, thereby inducing steep temperature drops within the rock matrix during the first days after commencing injection (see the dotted temperature contour lines in Fig. 3a and the constant temperature zones in 3b and 3c). Despite the reservoir rock continuously providing heat to the cooled region around the borehole wall, the rate of this heat transfer is insufficient to replenish the heat absorbed by the circulating fluid. Heat flow into the wellbore in deep CLGS is purely conduction-dominated and, thus, is controlled by the thermal conductivity of the rock, which typically presents values of 3 W/m/K in geomaterials. This low-thermal conductivity leads to the steep drop in temperature with time because the rock is not able to replenish the lost heat extracted by fluids at high-flow rates (limited conduction heat exchange), causing up to a 50 °C drop in production temperature in a time span of 12 days for one of our cases (for 25 kg/s-2 laterals in Fig. 3a). Higher production temperatures can be achieved by reducing the flow rate in the horizontal laterals. These dotted temperature contour lines from Fig. 3a get progressively pronounced with time and the existing low temperature regions reach farther parts of the rock matrix. These low temperature zones adjacent to the wellbore are not limited to the rock matrix near the end of the horizontal loop, but are developed almost along the entire length of the wellbore-rock contact zone (Fig. 3a, b, and c). The low temperature zones present a nearly parabolic temporal evolution (Fig. 3a), but, at any given instance of time, they are parallel to the fluid-rock interface if the multilaterals are perpendicular to the direction of gravity (Fig. 3b, c).

**Fig. 4:**
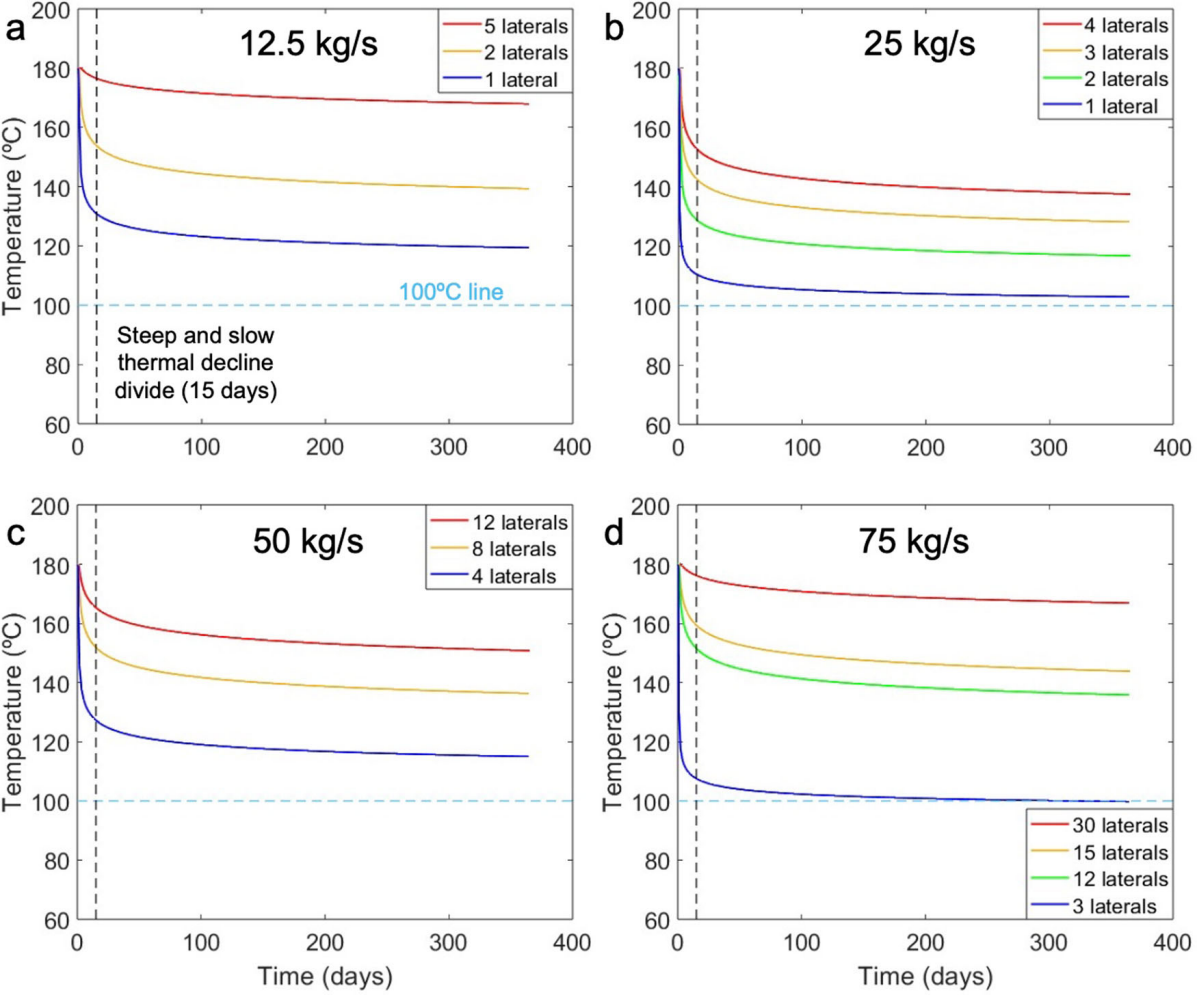
Temperature evolution curves for all our cases. Surface flow rates of **a** 12.5 kg/s, **b** 25 kg/s, **c** 50 kg/s, and **d** 75 kg/s, for different number of horizontal laterals. The black vertical dotted line at 15 days from the start of injection divides the steep and slow thermal decline regimes in all the curves. The light blue horizontal dotted line is drawn at 100 °C, indicating that when the curves fall below it, the case becomes incapable of generating electricity.

The inability of the fluid-rock interface to regain its ambient temperature and its thermal decline while fluids flow through it leads to a drop in the production temperature of water, which is very steep at first (50 °C in the first 12 days) and later tends to slow down (13 °C in the remaining 353 days (Fig. 3 and green curve in Fig. 4b). We can observe this shift between the steep and slow thermal decline regimes in Fig. 3, where the density of isotherms in the wellbore is high at the beginning, but subsequently declines (note the log scales in Fig. 3a). When the production temperature falls below 100 °C, we assume that the fluid becomes incapable of generating electricity even through Organic Rankine Cycle (ORC) based power plants (blue curve that goes below the horizontal light blue dotted lines in Fig. 4)^31^. This steep drop in temperature during the initial phase varies in magnitude (25 °C to 72 °C in the first 15 days) and is pronounced for all horizontal flow rates, except for those lower than 5 kg/s (red curves in Fig. 4a, c, and d). Out of the 14 considered cases, only three of them have production temperatures higher than 150 °C by the end of the first year (red curves in Fig. 4a, c, and d). The highest heat losses are observed for 12.5 kg/s-1 lateral, 25 kg/s-1 lateral, 50 kg/s-4 laterals and 75 kg/s-3 laterals (blue curves in Fig. 4). Interestingly, electricity cannot be produced for the 75 kg/s-3 laterals case (Fig. 4d). Hence, it is necessary to quantify these heat losses and the time intervals of their occurrence.

To optimize the number of horizontal laterals and total flow rates during the planning stage for maximum thermal recovery of the CLGS, the temperature drops associated with different horizontal flow rates should be quantified (Table 3). Temperature declines of 20–80 °C can be segregated into several time intervals of increasing number of days in Table 3. We observe temperature drops of 20 °C during the first 5 days of injection for our models with horizontal flow rates ranging from 6.25 kg/s to 8.33 kg/s. Temperature drops of 40 °C are common in the same interval for the horizontal flow rate of 12.5 kg/s (12.5 kg/s-1 lateral, etc.) in all our models (Table 3). Thermal losses of 60 °C–80 °C during the first year of injection are not rare in CLGS, which is not ideal as this is lost heat that could have been tapped (Fig. 4 and Table 3). In Table 3, if the production temperature of a model drops by 60 °C in the first five days, for example, 25 kg/s-1 lateral, it is only mentioned in the first column of the 60 °C row and not in 20 °C and 40 °C drop rows, and so on. The same holds true for the last column as well. If the production temperature of a model does not drop by 40 °C in one year, it does not drop beyond that by definition, and is not repeated in other rows of the same column.

**Table 3:** Summary of thermal losses in all our models

Temperature drop of/in number of days	0–5 days	5–10 days	10–15 days	15–30 days	30+ days	Does not drop in one year
20 °C	25 kg/s-3 laterals, 25 kg/s-4 laterals, 50 kg/s-8 laterals, 75 kg/s-12 laterals	12.5 kg/s-2 laterals	75 kg/s-15 laterals		50 kg/s-12 laterals	12.5 kg/s-5 laterals, 75 kg/s-30 laterals
40 °C	12.5 kg/s-1 lateral, 25 kg/s-2 laterals, 50 kg/s-4 laterals			25 kg/s-3 laterals	12.5 kg/s-2 laterals, 25 kg/s-4 laterals, 50 kg/s-8 laterals, 75 kg/s-12 laterals	50 kg/s-12 laterals, 75 kg/s-15 laterals
60 °C	25 kg/s-1 lateral, 75 kg/s-3 laterals				12.5 kg/s-1 lateral, 25 kg/s-2 laterals, 50 kg/s-4 laterals	12.5 kg/s-2 laterals, 25 kg/s-3 laterals, 25 kg/s-4 laterals, 50 kg/s-8 laterals, 75 kg/s-12 laterals
80 °C					75 kg/s-3 laterals	12.5 kg/s-1 lateral, 25 kg/s-1 lateral, 25 kg/s-2 laterals, 50 kg/s-4 laterals

**Fig. 5:**
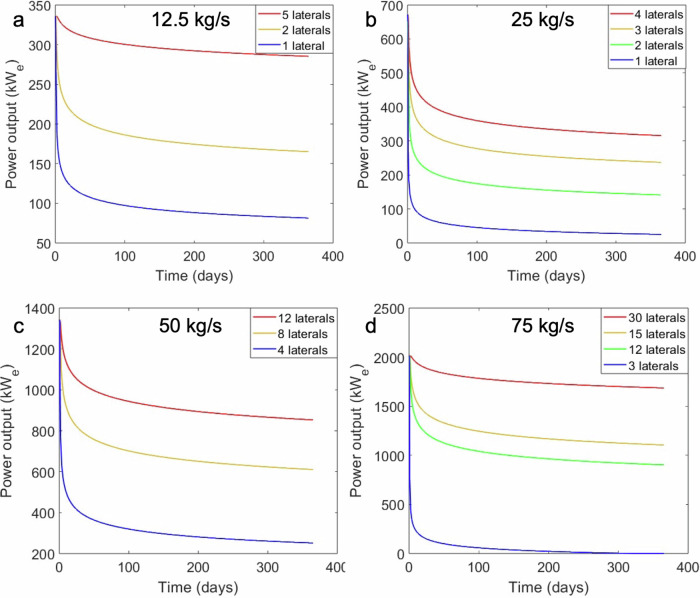
Power output (or electricity generation) capacity during one year for all our cases. Surface flow rates of **a** 12.5 kg/s, **b** 25 kg/s, **c** 50 kg/s, and **d** 75 kg/s. The color scheme of the curves is the same as in Fig. 4. The blue curve in (**d**) runs along the *x*-axis after a while because the temperature drops below 100 °C (as seen in Fig. 4), which makes it unsuitable for electricity generation.

The amount of electricity (power) that can be generated by CLGS is proportional to the production temperature, the electricity generation efficiency of power plants and the fluid flow rates (Eq. (3)). Owing to the huge temperature losses in the horizontal section of CLGS and the efficiency of power plants, it takes at least 15 laterals and a flow rate of 75 kg/s to stay above the megawatt of electricity mark by the end of one year in all the considered cases. Even though horizontal flow rates determine the production temperature in our cases, high total flow rates are more important for translating the high production temperatures into the magnitude of electricity that can be generated (red curves in Figs. 4a, 5a and 4d, 5d for the same horizontal flow rate). However, in a fixed subsurface setup (with a constant number of laterals), a higher total flow rate does not mean higher power output, but rather, a drastic drop in the amount of power output owing to the rise in thermal losses (e.g., for cases with 2 laterals in Fig. 5a, b, 3 laterals in Fig. 5b, d).

After the steep decline in power output during the first days, the power output curves (also production temperatures) relatively flatten out during the subsequent years. The power output at the end of the year, YEP as we define it, is the value that is consistently generated throughout the whole year, and this is the value that we consider for calculating the yearly revenue of the plant. Six of our models (one for each distinct horizontal flow rate) simulate thirty years of production to determine the temperature decline trend over the project lifetime. The decline trends could be identified by one or two straight lines in the log-time scale, and these lines are used to calculate the YETs beyond the first year until the last (Supplementary Fig. 1 and details in Supplementary Material). These YETs are used to calculate the yearly power over the lifetime of the project (30 years). The most promising of all our cases consistently generates above 1.5 MW_e_ in one year (red curve in Fig. 5d), but at the expense of an excessively high number of laterals: 30 (5 km each). The vertical drilling costs in EGS, according to a report published by the National Renewable Energy Laboratory (NREL), USA, in January 2023, decreased to 80% of the ideal case reported in the GeoVision report^32–34^. Applying that to our case of drilling a 4-km deep vertical wells would cost around $2.648 million. In the wide range of lateral drilling costs ($200/m to $600/m), which depend on bit lives and rates of penetration, we consider the average cost as $400/m for calculating the horizontal drilling costs for our cases^19^. Our horizontal laterals, being 5 km each (total horizontal length for the 30-laterals case is 150 km), are at a depth of 4 km (the total vertical drilling length is 8 km), so it adds up to a total drilling length of 158 km. Keeping the technical feasibility part of this aside, the drilling costs alone would exceed $65 million, which is immensely high (Table 4). Additionally, there is the capital cost of setting up an ORC-based surface plant that ranges from $2000/kW_e_ to $3000/kW_e_ (we use the average of $2500/kW_e_)^19^. Annual operations and maintenance costs account for 1.5% of the surface plant costs. Considering all these costs, the total lifetime costs of our most promising case add up to $72.60 million (Table 4). The summary of all the costs for our models as listed above is shown in Table 4.

**Table 4:** Total costs of all models (all costs are in $M)

Number of laterals (total flow rate (kg/s))	Total drilling costs (lateral $400/m)	Surface plant costs ($2500/kW_e_)	Lifetime OPEX (yearly OPEX is 1.5% of surface plant costs x 30 years)	Total costs (CAPEX + OPEX)
1 (12.5)	7.29	0.84	0.37	8.51
2 (12.5, 25)	9.29	1.68	0.75	11.73
3 (25)	11.29	1.68	0.75	13.73
4 (25, 50)	13.29	3.36	1.51	18.16
5 (12.5)	15.29	0.84	0.37	16.51
8 (50)	21.29	3.36	1.51	26.16
12 (50, 75)	29.29	5.04	2.26	36.60
15 (75)	35.29	5.04	2.26	42.60
30 (75)	65.29	5.04	2.26	72.60

The surface plant costs are calculated using the peak power that is produced by the highest flow rate in a fixed number of laterals setup (Fig. 5 and Table 4). The economic analysis of the 25 kg/s- 1 lateral case has not been shown as the production temperature drops below 100 °C in the first few years, making it unfit for electricity generation in the long term. The case with 75 kg/s-3 laterals is also not fit for long-term electricity generation as its production temperature goes below 100 °C within the first year (Figs. 4d and 5d). All the other 12 cases produce electricity for the assumed lifetime of 30 years. The surface plant cost and the corresponding OPEX over the lifetime of 12.5 kg/s-5 laterals are lower because we only have one flow rate for this lateral setup, and the power it produces is lower when compared to higher total flow rates like 25 kg/s and 50 kg/s (Table 4). Total costs or project expenditure of all our cases range from over $8 million to $72 million based on the number of laterals and the capacity of the surface plants (these depend on the total flow rates) (Table 4).

**Fig. 6:**
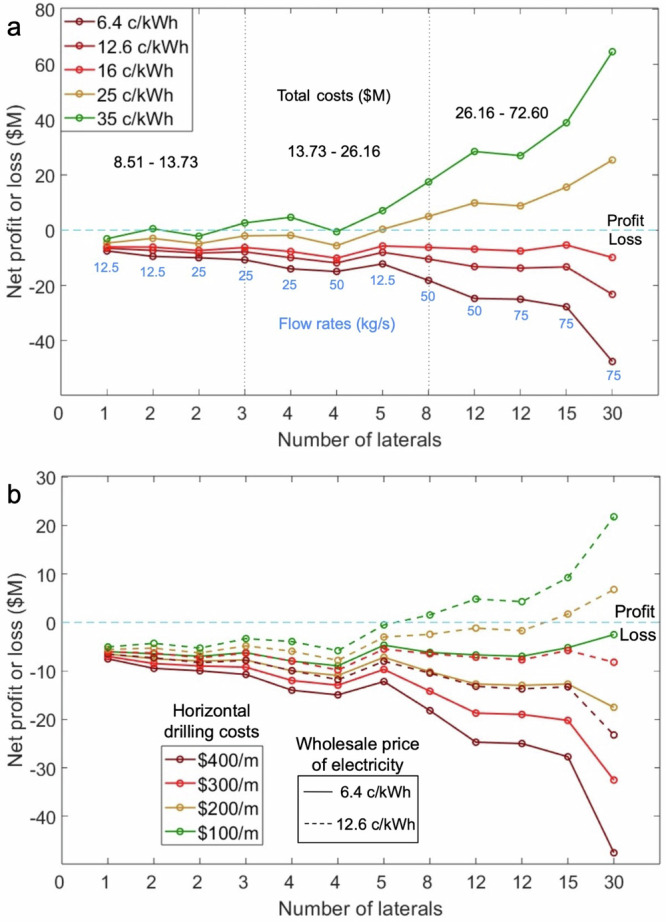
Net lifetime profit or loss curves for all viable cases. **a** Net profit or loss curve for 12 of our cases with different electricity prices for the base horizontal drilling price of $400/m. The black vertical dotted lines in (**a**) separate the total cost regions, indicated with black numbers. Flow rates corresponding to the laterals are shown in blue numbers. **b** Net profit or loss curves for all our cases with varying horizontal drilling costs and two wholesale prices of electricity. The blue horizontal dashed line separates profit and loss in both figures. Solid lines are the curves for 6.4 c/kWh and dashed lines are the curves for 12.6 c/kWh in (**b**).

Managing to get a profitable return on investment over these high project costs is difficult with the quantities of power produced by them. To compute the revenue generated by these cases, we use a range of documented electricity prices from the US markets. Wholesale price of electricity is the price at which energy generation companies sell it to the energy distributor. The highest wholesale price of electricity among selected regions of the US in November 2024 is 6.4 c/kWh, whereas the average retail price of electricity during the same month is 12.6 c/kWh^35,36^. However, some states on the east coast of the US sold electricity at a higher retail price of 25 c/kWh in the same month^36^. We also consider a case with a wholesale electricity price of 35 c/kWh to see how the revenue improves (Fig. 6a). The case producing the most power (75 kg/s-30 laterals) would be the most sensitive to changes in electricity pricing as observed in Fig. 6a, but even this case fails to generate profits by a margin of around $10 million with 16 c/kWh as the wholesale price (bright red curve in Fig. 6a). With a price of 25 c/kWh, we observe six cases (12.5 kg/s-5 laterals, 50 kg/s-8 laterals, 50 kg/s-12 laterals, 75 kg/s-12 laterals, 75 kg/s-15 laterals and 75 kg/s-30 laterals) registering profits of 0.33 (2%), 5 (19.1%), 9.86 (26.9%), 8.79 (24%), 15.57 (36.5%) and 25.37 (34.9%) million USD, respectively (yellow curve in Fig. 6a). Eight of our cases lead into healthy profits ranging from 2.6 million to 64 million USD at an exorbitantly high wholesale price of 35 c/kWh for the base horizontal drilling cost of $400/m (green curve in Fig. 6a).

Advances in drilling technology would reduce drilling costs, which might benefit CLGS as they require extensive drilling in their development. Taking this into account, we use four different lateral drilling rates- $400/m (base case), $300/m, $200/m, and $100/m to quantify their impact on the net profit of the cases (Fig. 6b). As the revenues from the wholesale price of 6.4 c/kWh are very low, we use another wholesale price of 12.6 c/kWh to assess what would happen by almost doubling the current wholesale price (solid and dotted lines in Fig. 6b). While the lateral drilling cost of $100/m is not sufficient to generate profits at 6.4 c/kWh for any case, 50 kg/s-8 laterals, 50 kg/s-12 laterals, 75 kg/s-12 laterals, 75 kg/s-15 laterals and 75 kg/s-30 laterals garner a profit of $1.54 million (5.8%), $4.81 million (13.1%), $4.27 million (11.6%), $9.22 million (21.6%) and $21.78 million (30%) at 12.6 c/kWh (dashed green curve in Fig. 6b). The inability to generate profits at competitive wholesale prices like 6.4 c/kWh even with drastic improvements in drilling costs is due to the rapid temperature drop caused by the cooling of the rock matrix surrounding the rock, which diminishes the real potential of the geothermal resources. Assuming that we can drill and complete 8 laterals successfully (total drilling length of 48 km) with a drastic drop in horizontal drilling costs to $100/m, we get a profit of just $1.54 million (5.8%) at a high wholesale price of 12.6 c/kWh (retail price to the consumer would be higher than this), which is a real challenge to achieve (Fig. 6b). All the scenarios beyond 8 laterals show profits with the highest one being $21.78 (30%) million for 30 laterals (Fig. 6b). Claims of universal geographical scalability of CLGS in power generation (not coupled with direct use application) at competitive prices are not supported by simulation results, which demonstrate a rapid temperature drop at the production well caused by limited heat conduction from the rock to the flowing water within the well.

## Discussion

### Steep drops in production temperature can only be prevented by drilling several multilaterals, which is excessively expensive

There are only two ways to counter the restraint imposed by steep temperature drops on CLGS—(i) long well shut-in to let the heat recover, or (ii) have a very low horizontal flow rate. Shutting in the well would mean stopping the production of electricity, which would make these systems capitulate the non-intermittent uniqueness of geothermal energy systems. Even if shutting-in recovered the heat in a short period, re-injection would drop the temperatures back within a few days, which is not a sustainable option in the long-term operation of a plant. The second option of having a very low horizontal flow rate is better because of the low heat extraction rate, which gives time for the heat from further sections of the rock to reach the fluid-rock interface and prevents the steep drop in temperature, as seen in the red temperature decline curves in Fig. 4a, d (horizontal flow rate of 2.5 kg/s per lateral). Having a high-production temperature is not enough to ensure high-magnitude power production, it also requires a high vertical (or total) flow rate in the system (Figs. 4a and 5a, 4d and 5d). The only way to have a low horizontal flow rate in a lateral for high vertical flow rates is to have several multilaterals in the subsurface (Fig. 5d). Among the considered cases, the most promising one has 30 multilaterals and a vertical flow rate of 75 kg/s, but still does not manage to economically breakeven by a margin of $47.52 million for a wholesale price of 6.4 c/kWh (Fig. 6a). Therefore, a reservoir temperature of 180 °C is not nearly enough to develop economically profitable CLGS for electricity generation (without coupling it with district heating) with the given subsurface setups. Apart from the huge cost of drilling several multilaterals, there is no project to date that has been able to successfully drill 10 s of kilometers in the subsurface.

### High drilling costs lead to very bad economics for developing CLGS solely for electricity generation in areas outside high geothermal gradients

Developing CLGS with limited multilaterals for electricity generation would technically make sense only in high-temperature reservoirs (>250 °C) as we end up with high-production temperatures (in the 160 °C–200 °C range, depending on the number of laterals and the flow rates) even after the drastic thermal losses in the horizontal laterals^11,19^. Higher temperatures also provide higher heat to electricity conversion efficiencies, while past data shows them to be in the single digits for binary plants below 160 °C^21^. Even in the high-temperature cases, it is necessary to have high surface flow rates and multiple laterals to maximize the profits from the project. Reaching high temperatures (>250 °C) at depths of 3–5 km is only possible in places with high geothermal gradients, which are localized in certain kinds of geologies, such as tectonic plate boundaries, near volcanoes and magma chambers^37^.

For CLGS to be truly scalable and economically profitable for electricity generation, they need to be able to reach these high temperatures anywhere on the planet. As the average geothermal gradient of the Earth is around 25 °C/km, and assuming a surface temperature of 15 °C, they need to drill to a vertical depth of 9.4 km to reach 250 °C and then drill several multilaterals, where the technical difficulty and cost of drilling only increases with temperature and depth. In our models, we reach 180 °C at a depth of 4 km in a region with a decently good geothermal gradient of 41.25 °C/km, and our best power-producing scenario (75 kg/s-30 laterals) requires a total project cost of $72.60 million and $27.60 million with lateral drilling costs of $400/m and $100/m, respectively. These cases generate a net loss of $23.22 million and a profit of $21.78 million at the wholesale price of electricity of 12.6 c/kWh, respectively (Fig. 6b). Even if the lateral drilling costs drop to $100/m, we would need a wholesale price of electricity higher than 12.6 c/kWh for many of our cases (more than 5 laterals) to generate profits. This drives the retail price of the end user to be much higher and would need heavy subsidies from the government to be competitively priced against fossil fuel-based electricity prices and other renewables. In regions with the average gradient, it would take 6.6 km to reach 180 °C, which would be economically worse than what our results show as there is an additional vertical drilling cost of 5.2 km. Owing to current technical limitations and very high-capital costs associated with drilling 10 s of kilometers underground combined with the drastic thermal losses for high horizontal flow rates, which is a major inherent physical limitation of CLGS, we can affirm that solely electricity generation from these systems is currently not scalable beyond regions of high-geothermal gradients^19^. However, coupling electricity production with large-scale direct use applications like district heating could increase the revenue of these systems and might possibly attain profits at scale^11^. This coupling with district heating becomes a geographic limitation, as there must be buyers of heat near the project site, which would also increase the risk to nearby communities or industries in case something goes wrong.

### Potential risks associated with CLGS and why fracture-based EGS is better?

The motivation to find an alternative way to harness geothermal energy beyond fracture-based EGS seemed to have risen after the *M*_w_ 3.4 earthquake at the Basel EGS project and accelerated after the *M*_w_ 5.5 earthquake triggered by hydraulic stimulation at Pohang EGS^6,7^. CLGS started to attract attention and investments as a viable alternative that does not generate induced seismicity during any stage of its development or operation because it does not involve hydraulic fracturing/stimulation^38^. The choice to avoid fracture-based systems for wellbore-sized laterals comes with a challenge of insufficient fluid-rock contact area for adequate heat extraction, which leads to the compulsion of drilling 10 s of kilometers of multilaterals to compensate for that^39^. This setup has the potential to influence a large reservoir volume in terms of both pore pressure (in case of open-hole horizontal laterals) and thermoelastic stress perturbations over long periods. Thermoelastic stresses are capable of inducing seismicity when cold fluids are injected into a very hot reservoir rock for prolonged periods, and it is well observed in the convection-dominated The Geysers geothermal field^40^.

Deep CLGS are more likely to be developed in stiff crystalline rocks with preferably a few or no fractures to have a cost-effective open-hole completion throughout the horizontal section^12^. If the seal/lining in the 10 s of km cased multi-laterals fail in fractured rock systems where one or more laterals intersect with fractures of fault zones, the entire CLGS would be compromised. Even if these systems encounter a few pre-existing natural fractures around the horizontal laterals or are developed close to a fault system, the highest susceptibility of reactivating them through thermal stress reduction is in a normal faulting stress regime (even for a temperature change of <25 °C) within a highly stiff rock matrix as the mechanical response to heat depletion is more pronounced in stiff rock^41^. Heat depletion or cooling in stiff rock around the multilaterals could lead a considerable reservoir volume contraction (30% of heat drainage volume) and shear failure of the inherent fractures within the assumed 30-year lifetime of the CLGS^41^. This thermal stress reduction can also affect the stability of nearby or distant critically stressed faults (up to 100 s of meters away)^42,43^. As CLGS need to target high-temperature reservoirs to maximize revenue, this shows that there is an inherent risk of inducing microseismicity due to cooling-induced thermal stress reduction and this risk escalates with increasing temperature difference between the reservoir and injected fluids (note that if thermal applications are added to electricity generation to increase the project revenue, the re-injection temperature would be lower), which challenges the popularly preconceived notion that this technology is risk-free.

The occurrence of relatively high-magnitude earthquakes in subsurface engineering in recent years (due to, e.g., wastewater injection, underground gas storage and EGS) demanded a better understanding of the causal mechanisms of these induced earthquakes and the key factors controlling their maximum magnitude^44–48^. The main advantage that fracture-based EGS has over CLGS is the large contact area with the rock through hundreds of small fractures^17^. The high number of fractures and the fracture apertures being so small divide the surface flow rate into very small flow rates within fractures. This allows the heat from further sections of the rock matrix to replenish the extracted heat and leads to a very slow thermal decline, which improves the longevity of the project^17,49,50^. The EGS project at Soultz-sous-Forêts, France, has been consistently generating 1.7 MW_e_ of electricity since 2008 with an average flow rate of 30 l/s with a reservoir temperature of 165 °C^51,52^. Whereas, the best of our considered cases can generate around 1.68 MW_e_ during the first year, but at an expense of $72.60 million and 30 laterals. The low capital cost of fracture-based EGS, when compared to CLGS, along with the slow thermal decline, makes it economically profitable and sustainable over long periods. This has recently been demonstrated in projects by Fervo Energy at Nevada (Project Red), where no thermal decline was recorded in 6200 h (258 days) and Utah (Project Cape), where a 30-day circulation test showed no sign of thermal decline for a sustained output of 8–10 MW_e_^53^. These systems by Fervo Energy were developed by propping hydraulically created fractures rather than hydro-shearing existing ones, which seems to be a promising way forward. Improvement in drilling time and costs displayed at projects by Fervo energy and FORGE project in Utah, along with multi-stage stimulation to restrict induced seismicity show great promise for future EGS projects^54^. Hence, cautious development of fracture-based EGS is more convenient than CLGS to truly scale up geothermal energy-based large-scale electricity generation around the globe.

## Conclusions

The results of our study explain in detail what happens in the rock matrix when fluid flows through a cased vertical well and an open-hole section of the horizontal laterals in a span of one year and how it consequently influences the production temperature and the revenue of the closed-loop geothermal project. The main highlights of our work are:

i. High-horizontal flow rates in CLGS lead to steep and rapid temperature drops in the rock matrix because the low thermal conductivity of the rock is unable to replenish the lost heat fast enough. The only way to have a high-power production potential is to ensure low- horizontal flow rates by drilling several multilaterals.
ii. Low-horizontal flow rates result in a higher production temperature in CLGS, but high total flow rates are required to generate more power.
iii. The calculated revenues from 12 of our cases (reservoir temperature of 180 °C) for a range of vertical and horizontal flow rates were unable to recover the 30-year lifetime costs of the projects by margins ranging from $2.52 million to $8.95 million, even with a lateral drilling cost of $100/m (at a wholesale price of 6.4 c/kWh). A profit of $21.78 (30%) million can be generated with a high wholesale price of 12.6 c/kWh, but at the expense of a total drilling length of 158 km and a project cost of $27.60 million (at an ambitious lateral drilling cost of $100/m).
iv. Claims of universal geographical scalability of CLGS in power generation (not coupled with direct use application) at competitive prices are not supported by simulation results, which demonstrate a rapid temperature drop at the production well caused by limited heat conduction from the rock to the flowing water within the well to be a limitation of this technology.

## Supplementary information

Supplementary material

## Data Availability

The data files of the numerical models can be accessed through the following link- 10.20350/digitalCSIC/16567.
