# Supplementary material for: On the limitations of closed-loop geothermal systems for electricity generation outside high-geothermal gradient fields
Source: Commun Eng. 2025 Jul 1;4:116. doi: 10.1038/s44172-025-00458-7 (PMC12219759; doi:10.1038/s44172-025-00458-7)
Supplement: Supplementary file 1 — Supplementary material [file 44172_2025_458_MOESM1_ESM.docx]

**Supplementary Material**


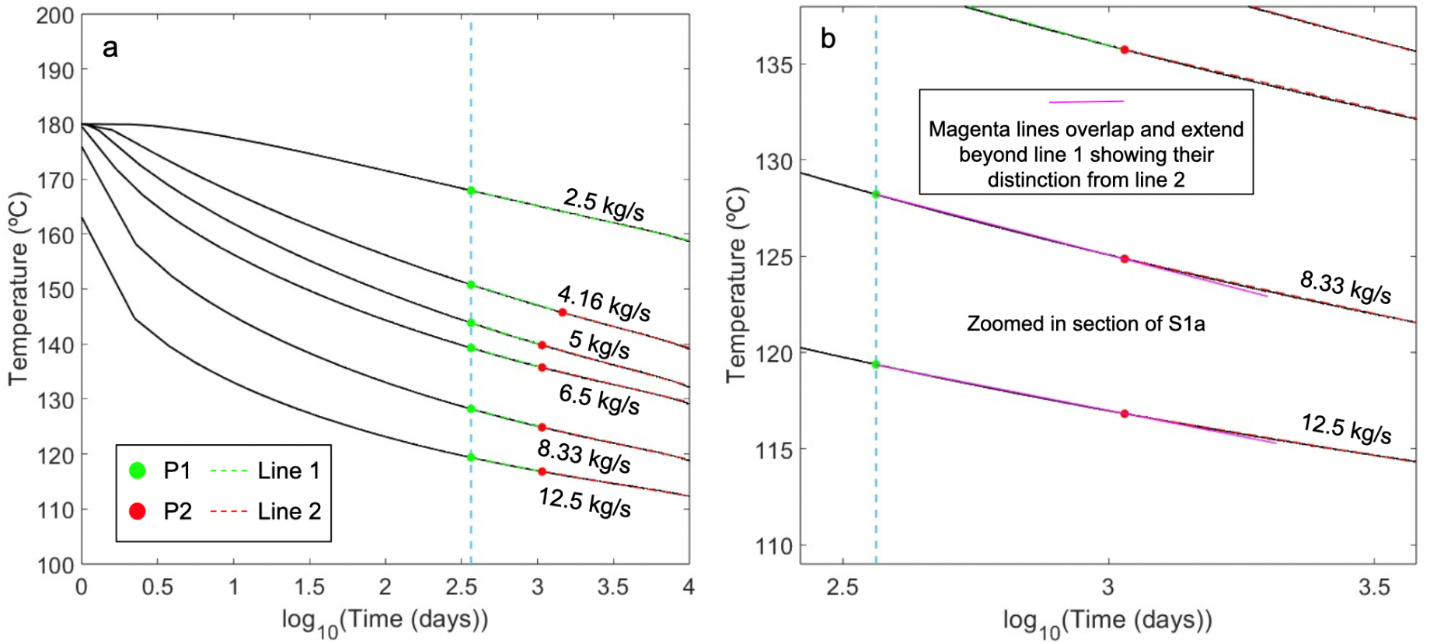


**Supplementary Figure 1.** **(a)** Temperature decline curves over the 30-year lifetime for every horizontal flow rate. Note that the x-axis is in log scale and starts at day 1 of water circulation as the earlier figures show detailed temperature decline until year 1 (indicated with the vertical blue dashed line). The green and red dashed lines over the temperature curves are used to calculate Year-End Temperatures (YETs) (details in Supplementary Table 1). **(b)** Zoomed in section of (a) showing the distinction between line 1 and line 2 for a couple of temperature curves.

We run six horizontal models (one for every distinct horizontal flow rate) for the project lifetime of 30 years and their temperature evolution curves are shown in Supplementary Figure 1 (note that the time is in log scale). The temperature decline of each model is computed based on its horizontal flow rate, as depicted in Supplementary Figure 1, and taking into account the injection temperature, which depends on the BHT of the corresponding vertical model. Beyond the one-year mark indicated by the blue vertical dashed line, the temperature trends in log time are linear, which fit in one (for flow rate of 2.5 kg/s) or two distinct lines (for the rest of the models) as shown by the green and red dashed lines over the temperature curves (Supplementary Figure 1). The starting points and slopes of these lines are given in Supplementary Table 1 (x-axis values in Supplementary Figure 1 are in log scale of days and values of time in Supplementary Table 1 are in years). The temperature evolution curve of 2.5 kg/s fits in a single line starting at one year until the end and has just one point and slope (Supplementary Table 1).

**Supplementary Table 1. Long-term temperature decline data.**

| **Horizontal flow rate (kg/s)** | **Time (years)/ Temperature (ºC) at P1** | **Time (years)/Temperature (ºC) at P2** | Slope of line 1 (green dashed lines in Figure S1a) | Slope of line 2 (red dashed lines in Figure S1a) |
| --- | --- | --- | --- | --- |
| 2.5 | 1/167.91 | - | -6.3380 | - |
| 4.16 | 1/150.787 | 4/145.759 | -8.3398 | -7.6861 |
| 5 | 1/143.84 | 3/139.794 | -8.4797 | -7.7340 |
| 6.25 | 1/139.296 | 3/135.730 | -7.4602 | -6.5861 |
| 8.33 | 1/128.216 | 3/124.861 | -7.0184 | -6.1376 |
| 12.5 | 1/119.374 | 3/116.813 | -5.3592 | -4.6854 |

The difference in slopes of both lines (Supplementary Table 1) is shown by extended magenta lines in Supplementary Figure 1b, which highlight that the correct fit require the second lines as the first ones deviate from the temperature trend beyond point P2 for five of our models (Table S1). These lines are used to calculate the YETs from the end of the first year to the assumed lifetime of 30 years. These YETs are used to calculate the YEPs, and the sum of all YEPs over thirty years results in the cumulative power generated by a specific subsurface setup, which is used to calculate the lifetime revenue generated.
